# Novel 3D composite for efficient photocatalysis in environmental remediation

**DOI:** 10.1038/s41598-024-68840-7

**Published:** 2024-12-23

**Authors:** Indra Sulania, Ranjeet Kumar Karn, Jacek Fiutowski, Till Leissner, Arkadiusz Goszczak, Elham Chamanehpour, J. A. Ajani Lakmini Jayarathna, Yogendra Kumar Mishra

**Affiliations:** 1 Materials Science Group, Inter University Accelerator Centre, New Delhi, Delhi 110067 India; 2 Department of Physics, Jamshedpur Cooperative College, Jamshedpur, 831001 India; 3 Mads Clausen Institute, NanoSYD, University of Southern Denmark, Alsion 2, 6400 Sønderborg, Denmark

**Keywords:** Metal oxide, Polyethylene glycol, ZnO tetrapods, Filler–polymer 3D composites, Environmental remediation, Environmental sciences, Materials science

## Abstract

Highly porous, self-supported 3D interconnected network-based nanomaterials hold immense promise in revolutionizing the field of catalysis. These materials combine two critical features; a large accessible surface and an overall active surface that leads to substantial catalytic effects. In this study, we developed a novel class of 3D composite material composed of zinc oxide tetrapods (ZOT) and polyethylene glycol (PEG) polymer, specifically designed for photocatalysis. A polymer composite of ZOT with PEG has been synthesized with 2.5 wt.% ZOT powder mixed with a PEG solution forming a 3D electrode. A consistent composite solution was obtained using probe-sonication and its thick layers were deposited on various substrates using the spin coating technique which were subsequently characterized for optical, morphological, and structural properties. The catalytic response of the fabricated 3D composite was evaluated both in solution and thin film forms under UV exposure. The surface-engineered ZOT-PEG composites showed an excellent capability to degrade the methylene blue (MB) dye in different forms under UV and normal light, opening their potential scopes in environmental remediation.

## Introduction

The escalating pollution of water bodies, due to untreated wastewater from the textiles industries and households being thrown into the natural water sources, poses a severe threat both to human health and the natural environment^1^. In fact, nearly ten thousand harmful organic dyes are being dumped or thrown into the water resources primarily from the textile manufacturing units which is becoming a matter of greater concern for aquatic lives and ecosystem too^2,3^. Materials based catalysis has attracted much attention over the past few decades owing to its potential applications in environmental remediation. Due to the emphasis on the chemistry behind the synthesis of various catalysts, it was earlier considered as the chemical field of study but recently, it has evolved into a multidisciplinary field of materials science and engineering^4^. Catalysis finds applications in a wide variety of fields such as mineral resources, green chemistry, fine chemicals and pharmaceuticals productions, hydrocarbons processing, wastewater treatment and water cleaning, gas pollutant emissions control, photo-electrochemistry and fuel cell technologies, and numerous other agriculture, food, energy, and environmental applications^1,5^. For decades, several conventional methods such as sedimentation, filtration through chemicals and membrane technologies have been used to eliminate the degradation-resistant pollutants such as harmful dyes or hazardous chemicals^6–8^. As advancement to tackle this challenge, the photocatalytic techniques have drawn much attention due to their high efficiency and low toxicity in decomposing organic contaminants under light exposure, thus, providing green and eco-friendly solutions^8^. Several photocatalysts such as CdS, TiO_2_, Fe_2_O_3_, ZnO, SnO_2_, and many more, have been widely studied for wastewater treatment^9–12^. However, few catalysts like TiO_2_ show a limited efficiency due to its wider bandgap and higher electron–hole recombination^9,10^. Therefore, developing the highly efficient light-driven catalysts for degrading the organic chemical pollutants is extremely important.

In addition, it is well known that photocatalyst powders are usually used during wastewater purification. However, it remains a challenge to collect these powders from the treated waters which lead to re-contamination in the natural water bodies. Consequently, developing the stable and highly efficient photocatalytic materials is in much demand to meet the present challenges without leaving the remnants. Several studies have shown that composite materials may turned out to be better catalysts by improving the charge separation and transfer phenomenon. Copper oxide (CuO), zinc oxide (ZnO) and titanium dioxide (TiO_2_) are wide bandgap semiconductors and may function as potential photocatalysts^9,10,13^. In particular, the ZnO semiconductor is widely investigated for applications in dye-sensitized solar cells because it exhibits a high bulk electron mobility with a band gap of ~ 3.37 eV and a large exciton binding energy of ∼60 meV^10,11,14^. It has gained a lot of interest because of advances in synthesis techniques in achieving hierarchical shapes as per specific applications such as photocatalysis due to its high photosensitivity and stability^15^. ZnO material has shown continued significance in pollution remediation applications owing to its unique photophysical and physicochemical properties which offers many advantages over other materials^3^. Moreover, the capping of polymers onto the surface of ZnO results in a combination of electro-chemical interaction, hydrogen bonding and Van Der Waals force^16^. Polyethylene glycol (PEG) is a non-toxic and highly soluble pore-forming agent^17^, which occupies space in the agglomerated materials such as ZnO and, thus, could potentially be used to modify the properties of various photocatalysts by affecting the shape, size and crystal structure of the host semiconductor materials^18^. Few reports suggests that due to different molecular weight of PEG, the size of nanoparticles may be manipulated for proper growth of the photocatalyst material which can improve the overall catalytic actions^8,19–22^. For a nonionic polymer like PEG, the hydrogen bonding is the primary mode of interaction with ZnO^16,23^. Recently, the growth of a novel 3D geometrical shape of ZnO nanostructure, i.e., tetrapod has been reported^24^ which offers multiple advantages of utilizing the nanoscale features in a very simple manner in contrast to conventional spherical and 1D/ 2D ZnO nanostructures. In the last ten years, studies on ZnO photocatalysts have been steadily increasing. The novel 3D geometry of tetrapods, in the form of interconnecting four 1D nanorods via a central crystalline core at an angle of around 110°, enables their potential use as fillers in the composites without any agglomeration due to their self-assembly feature. They have been utilized for multifunctional properties and applications^22^.

**Figure 1:**
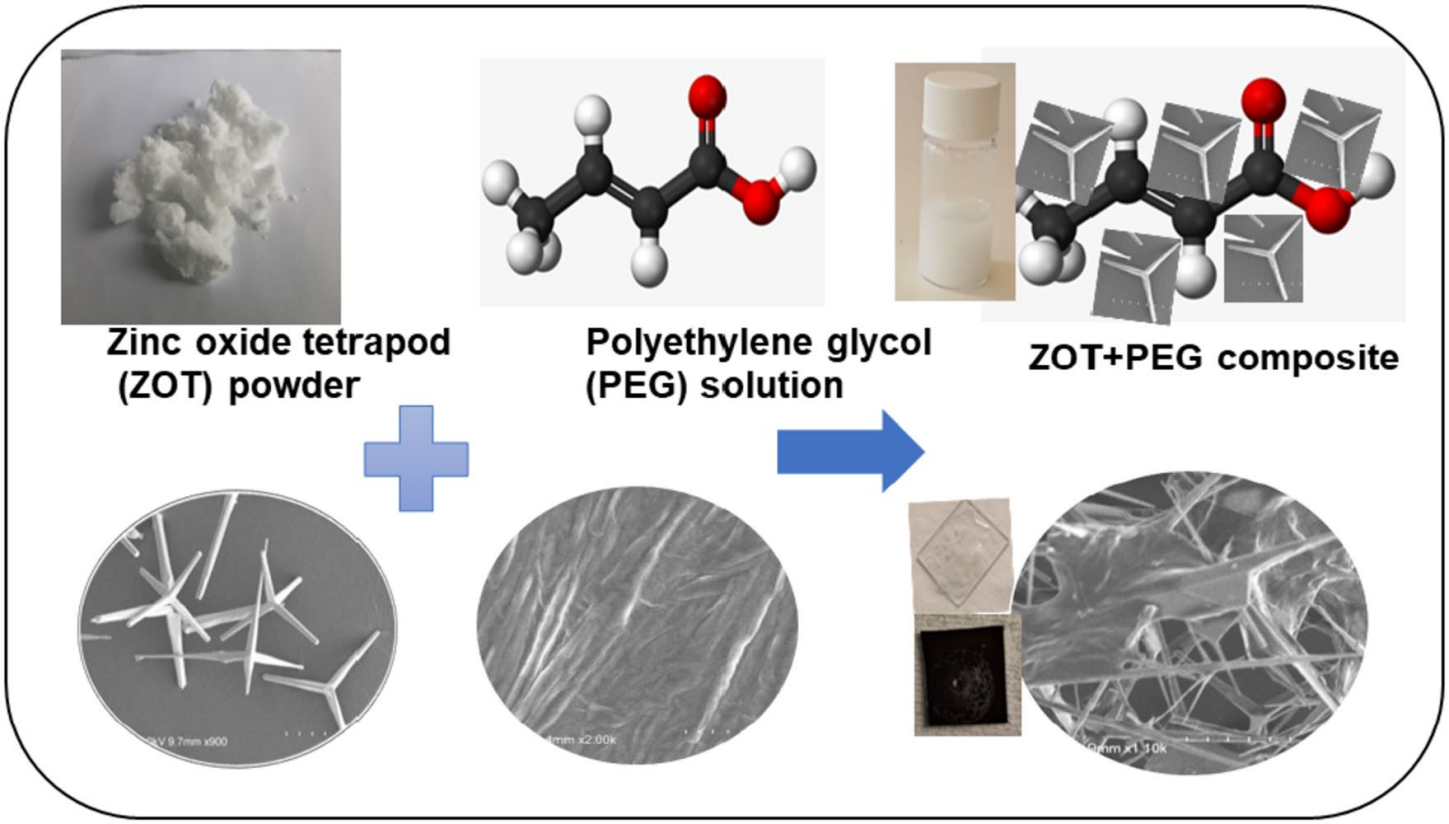
Schematic representation of ZOT incorporated with PEG polymer and actual representation with SEM images of ZOT and PEG to form the composite material.

Due to the unique features of ZOT, we introduced a novel 3D composite synthesized with ZOT and PEG as shown in the schematic in Fig. 1, as they hold immense importance to environmental monitoring^25^, and are efficient catalysis material due to its non-toxicity and high solubility. Thus, in the present study, thicker films of composite of PEG and ZOT have been formed on different substrates using chemical routes and characterized for their morphological, optical and structural properties. Their performance has been recorded as a catalyst for organic dye degradation. Such materials exhibit higher potential than the conventional powders used for catalysis due to their higher efficiency and bio-degradable nature for wastewater treatment, thus, acting as an efficient environmental remedial.

## Materials and methods

A pure polyethylene glycol polymer solution Hybri-Max™ was procured from Sigma-Aldrich. The powder of ZnO tetrapods (ZOT) synthesized using the flame transport synthesis (FTS) technique^17,18^ was used to form the composite of ZOT and PEG due to its non-toxicity and high solubility using the chemical routes. The solution of PEG polymer with ZOT powder was prepared in a glass bottle and ultra-sonicated for 30 min followed by probe sonication for 30 s at 20 W power to form a well- dispersed uniform solution. The sonicated solution was drop-casted on different substrates (Si, glass and CaF_2_) with a 20 µL pipette. Further, spin-coated at 1000 rpm for ~ 5 to 10 layers to form a thick composite layer and then dried on a heater plate at 110 °C for 30 min. The polymer composite samples were characterized with a surface profilometer from Dektak XT, Bruker, for the thickness measurements, and samples were found to be ~ 164 µm thick. Due to thicker samples, the samples were observed under a high-end Optical Microscope from Keyence, dual-objective zoom lens (20 × to 2000x) VH-ZST, USA, to study the surface uniformity of the composite layer at 500 to 2000 magnification. Further, their morphologies and composition were analyzed using a Hitachi S-4800 Scanning electron Microscope (SEM) with EDX performed at an electron energy of 10 keV. The detailed morphology of the composite sample was mapped using a He ion Microscope (Orion Nanofab, Zeiss) at a beam energy of 30 keV. The contact angle measurements on the composite sample were performed using DSA 10 from Kruss, Germany. The samples were characterized with a Raman spectrometer with an excitation wavelength of 532 nm in the 200 to 1350 (cm^-1^) wavenumbers range using an Alpha 300 Raman spectrophotometer from WITec, Germany, for the structural information and characteristic Raman shifts. The absorption properties of the composite samples were analyzed using an FTIR spectrophotometer, IRAffinity-1S from Shimadzu, USA for PEG and ZnO functional groups in transmittance mode from 400 to 4000 cm^−1^. UV visible absorption spectra were measured using a UV-2700 spectrophotometer from Shimadzu, USA in the 200 to 700 nm range. Methylene blue (MB) (from Sigma Aldrich) dye solution (0.1 mM) was used as a contaminant in deionized water. Its photocatalytic degradation for different exposure times under a tungsten UV lamp (~ equivalent to one sun intensity) in the presence of catalysis solution and electrode/ films were recorded using UV–Visible spectroscopy in a quartz cuvette, and their performance was compared. The synthesis and characterization of the samples were performed at the University of Southern Denmark (SDU), Denmark and Inter University Accelerator Centre, New Delhi, India.

## Results and discussion

### Morphological investigations

**Figure 2:**
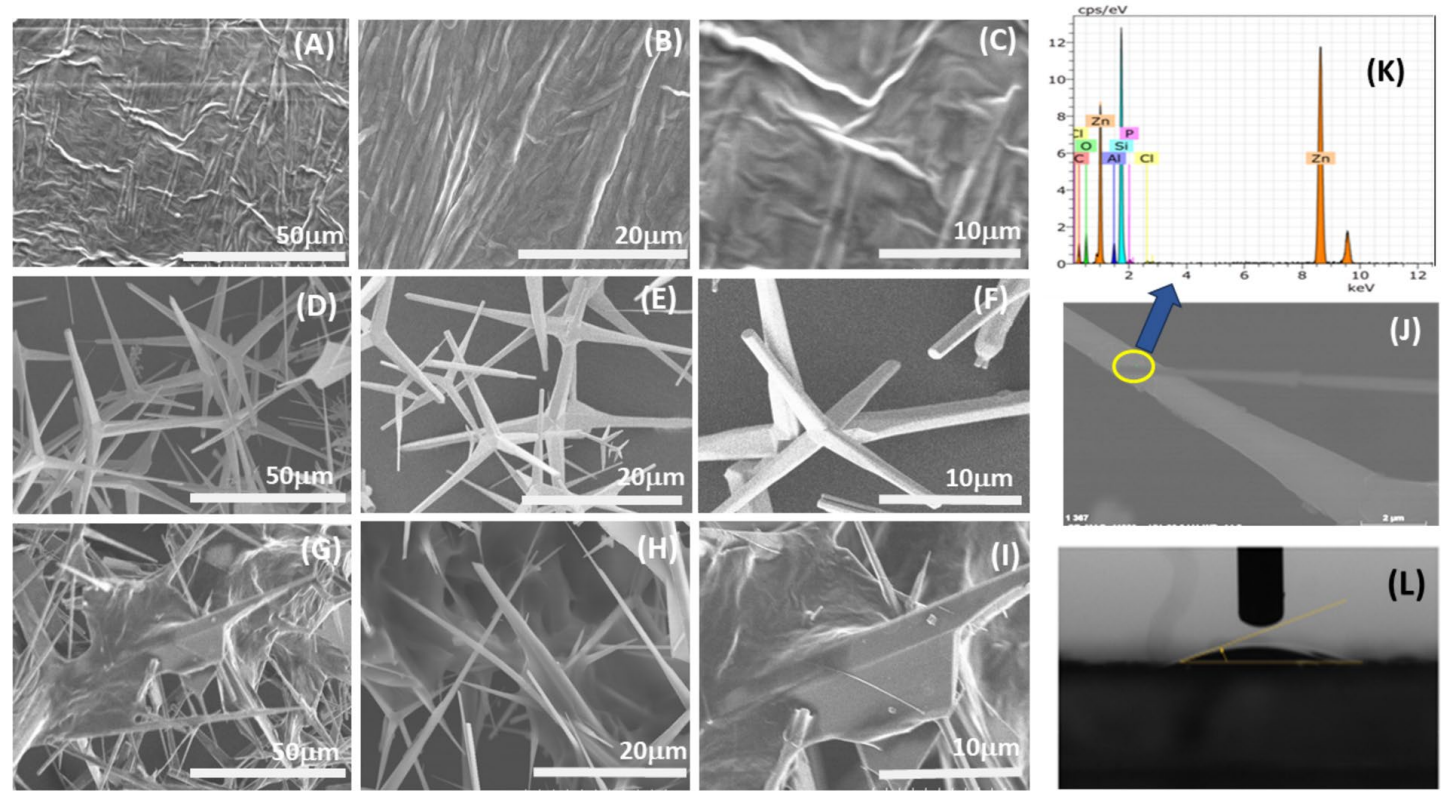
(**A**, **B**, **C**) SEM image of the polyethylene glycol at increasing magnifications; (**D**, **E**, **F**) Representative SEM images of zinc oxide tetrapods at various magnifications; and (**G**, **H**, **I**) SEM images of the fabricated ZOT-PEG composite sample at different magnifications; (**J**, **K**) The EDX analysis on the selected tetrapod leg, confirming the elemental compositions; (**L**) contact angle measurement of the composite sample with water droplet.

The morphological studies of the ZOT, PEG and (ZOT + PEG) composite deposited on Si were studied with SEM followed by EDX performed on one of the legs of the zinc oxide tetrapod. The SEM measurements in Fig. 2A,B,C show images of PEG polymer at different scan sizes, exhibiting the polymer morphology. The SEM images of ZnO tetrapod’s reveal an interconnected 3D network of the ZOT in Fig. 2D,E,F. The SEM images corresponding to fabricated ZOT-PEG 3D composite sample are shown in Fig. 2G,H,I which reveal that polymer exhibit an almost uniform distribution of ZOT filler elements. The EDX mapping was performed on one of the selected tetrapod’s leg and is presented Fig. 2J,K for the compositional information. The dimension of the tetrapods lies between 10 to 50 µm, with each leg arranged at ~ 110° connected with each other via central crystalline core to form a tetrapodal geometry, thus, forming a homogenous ZOT network filled with PEG polymer in form of 3D ZOT-PEG composite. The special 3D shape introduces extraordinary mechanical and optical properties in the composite^24^. The legs of the tetrapod’s are approximately 1 µm and 20 µm long, getting tapered at the ends in some of them.

The EDX investigations confirmed the presence of Zn with other elements like C and O suggests the capping of PEG on ZOT with PEG more densely gathered around the center of the tetrapods. The PEG accumulation around the central core, is a very common feature mainly due to the wrinkled morphology at the center of ZnO tetrapods. The contact angle of the (ZOT + PEG) composite film deposited on Si was calculated using DSA 10 software using tensile fitting. The contact angle of the film was found to be ~ 12 ± 0.1° with water droplet as shown in Fig. 2L, which shows the super-hydrophilic nature of the samples^17,26^ Since both, the ZnO and PEG exhibit inherent hydrophilic natures, the low contact angle obtained with respect to fabricated composites is very obvious.

**Figure 3:**
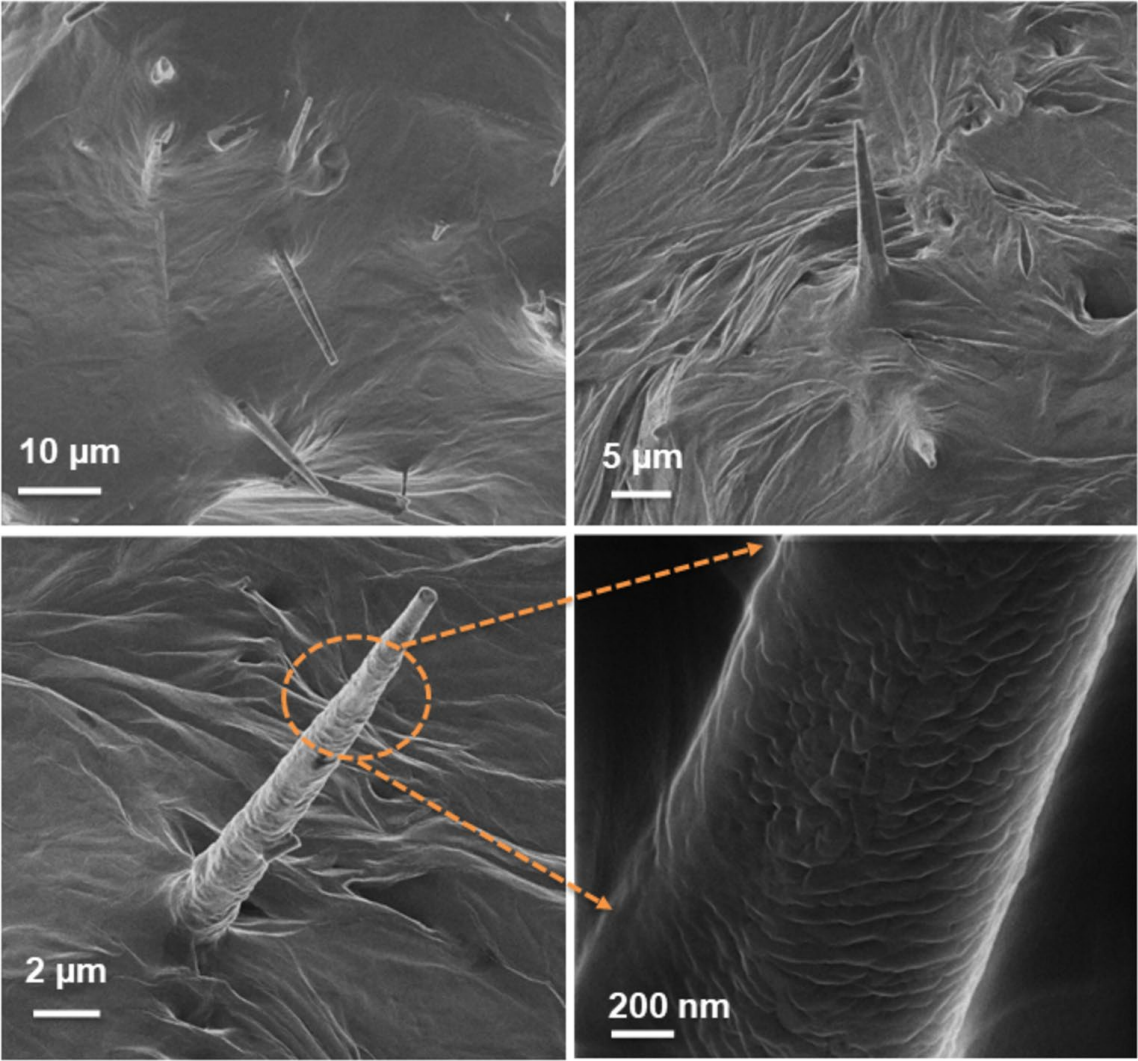
Helium Orion microscope images of the composite samples at different magnifications. The bottom row highlights a typical ZOT arm embedded in PEG and corresponding magnified area showing the typical morphology of the tetrapod legs in the composite.

To further confirm the capping of PEG on the tetrapods, a Helium-Ion microscope was used to analyze the samples in detail for high-resolution images and corresponding morphologies are shown in Fig. 3 at different scan sizes. The highest resolution image at 200 nm shows the proper capping of PEG on one of the tetrapod legs exhibiting a rough surface, further, confirming the findings of the EDX results.

### Optical and IR response of ZOT-PEG 3D composites

**Figure 4:**
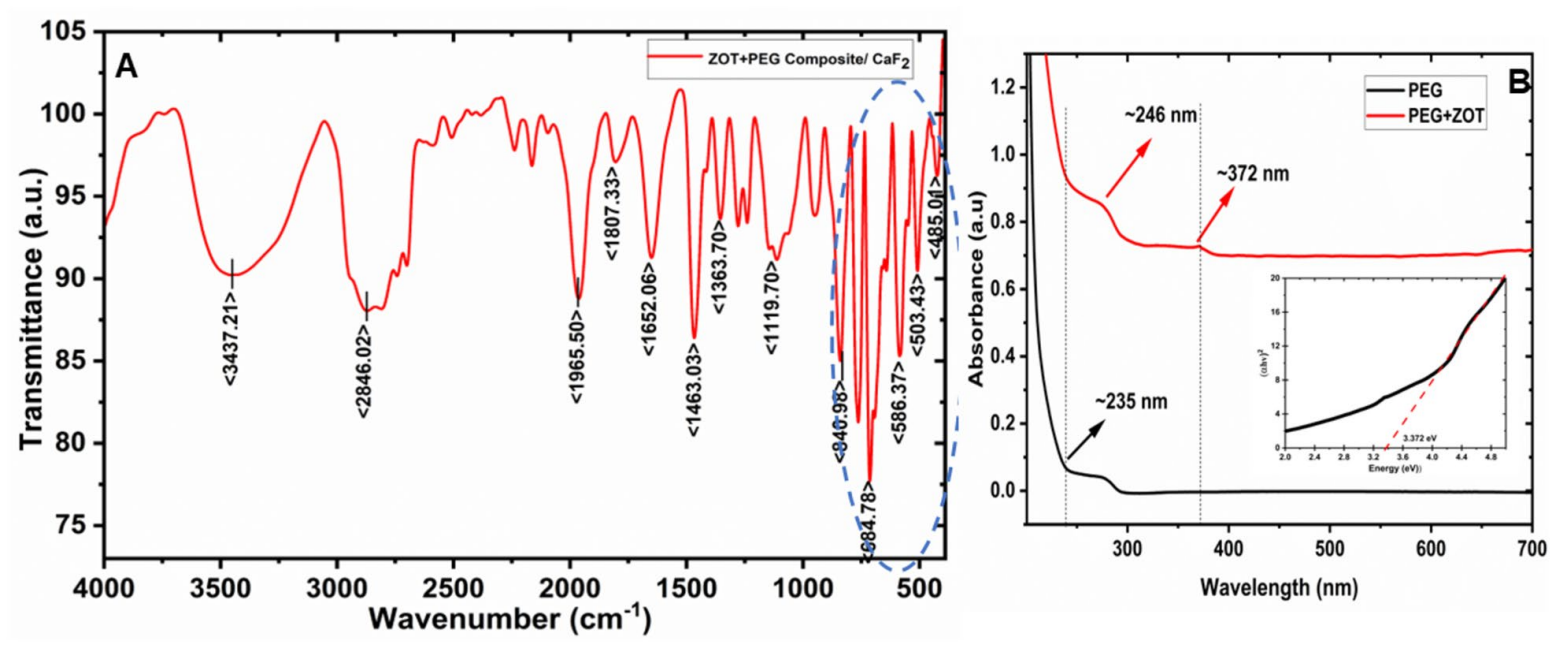
(**A**) FTIR and (**B**) UV visible spectra of the polymer composite of PEG and ZOT (with inset showing the Tauc plot for bandgap).

FTIR spectrophotometer provides qualitative information about the bond nature of adsorbed PEG molecules and the ZnO surface. The films deposited over CaF_2_ were used for these measurements to obtain the spectrum. At first, the background was taken with a bare CaF_2_ substrate, followed by the composite film. The spectra were recorded over a large range of 4000 to 400 cm^−1^ to scan the functional groups for PEG and ZnO, as shown in Fig. 4A. The peak between 3200 and 3600 cm^-1^ suggests the presence of a strong (O–H) hydroxyl group, however, it is shifted to a lower wavenumber ~ 3437 cm^−1^, which may be due to the formation of a hydrogen bond at the ZnO-PEG interface^27,28^ due to capping of PEG over ZOT^29^.

However, the characteristic sharp peaks of Zn–O at 685, 585, 503 and 465 cm^−1^ are visible, with splits in the presence of PEG (as highlighted in the spectra)^27,28^. Table 1 shows the stretching and bending of various functional groups present in the composite material. As evident in the FTIR spectra, many peaks with lower transmittance are visible, however, only major ones are indexed here. The strong C–O stretching peak observed between 1463 and 1692 cm^−1^ and the asymmetric stretching of the C–O between 1300 and 1390 cm^−1^ were also detectable. A peak at around 840 and 890 cm^−1^ indicates C-H groups from PEG. The peak at lower wavenumbers indicates the Zn–O at 460 to 685 cm^−1^ ^28^.

**Table 1:** Functional groups present in the ZOT-PEG composite sample.

Functional group	Wavenumber (cm^−1^)			Bending
OH	3437	2846	1965	(Symmetric stretching)
C–O–C	1652	1463	1360	(Asymmetric/ symmetric stretching)
C–H	892	840		Bending
Zn–O	685	586 503	465	Stretching (metal oxide)

The absorbance spectra were recorded using the UV visible spectrophotometer in the 200 to 700 nm wavelength range, as shown in Fig. 4B. The absorption band corresponding to PEG is visible at ~ 235 nm^27^. With the incorporation of ZOT, the absorbance shifted to a higher wavelength, due to the bond formation between Zn and H, and the peak corresponding to ZnO is visible at ~ 372 nm, shifted from its original position of ~ 376 nm^30,31^. The results complement the observations from FTIR. The higher wavelengths do not show any absorbance, clearly revealing the absence of any other contaminant in the sample. The bandgap of the composite sample has been analyzed using the Tauc plot at shown in the inset of the UV visible spectra by using Tauc’s plot method from equation:

1 $$\left( {\alpha hv} \right)^{n} = \, A\left( {hv \, - \, E_{g} } \right)$$

where α is the optical absorbance, hv is the incident photon energy, n can be 2 and 1/2 for allowed direct and allowed indirect transitions, respectively, A is a constant, and E_g_ is the energy band gap of the semiconducting material^31^. The calculated bandgap of the sample was found to be ~ 3.372 eV (almost close to bulk zinc oxide).

### Confocal Raman studies of ZOT-PEG composites

**Figure 5:**
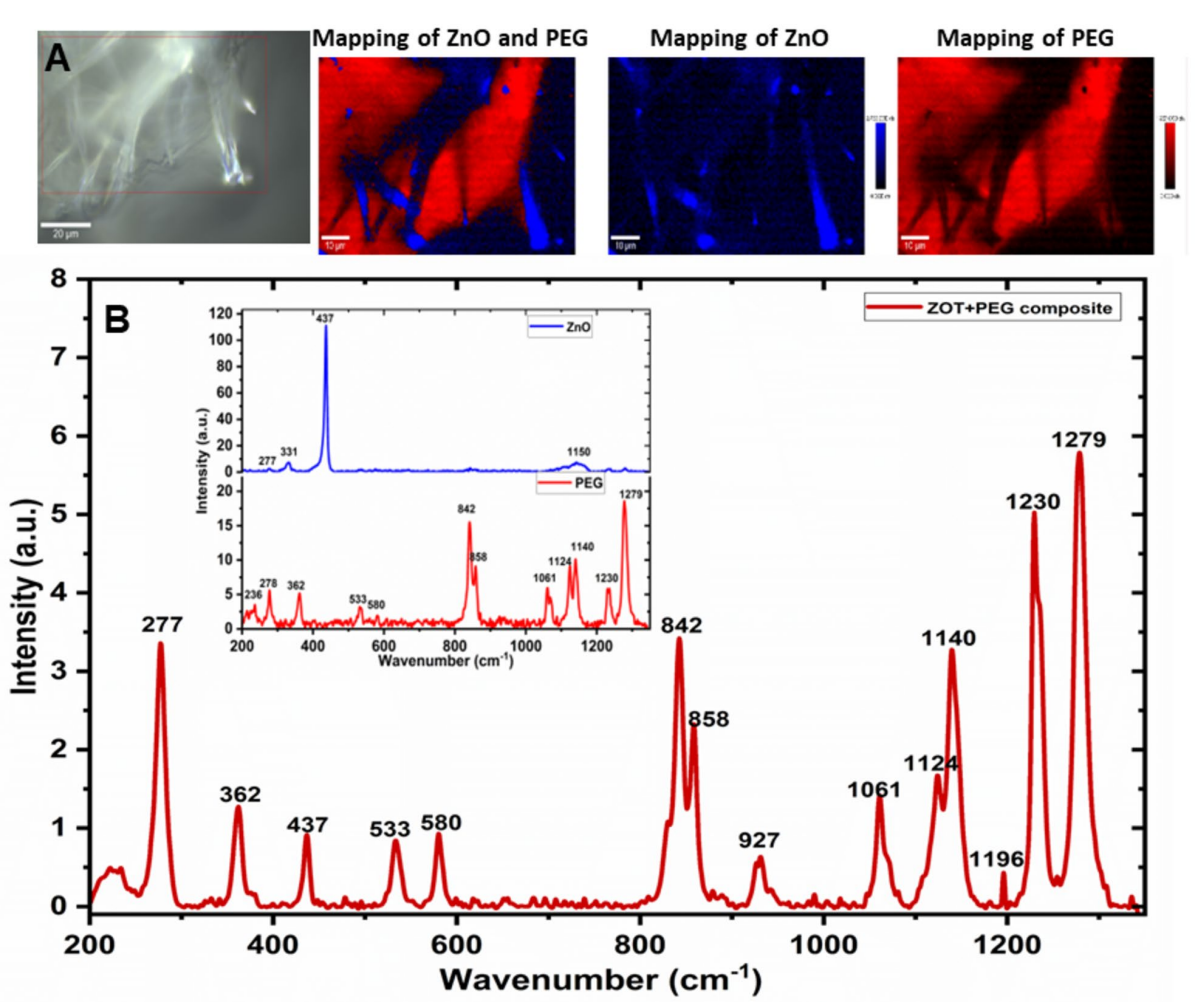
Confocal Raman spectroscopy of the composite film; (**A**) Images show the mapping of the ZOT (blue) and PEG (red) and the area where the spectrum is taken; (**B**) Raman spectra for the composite sample and inset shows the deconvoluted spectrum of ZnO and PEG.

Raman scattering is one of the major tools for identifying the chemical structure of the materials. The Raman measurements were performed in the confocal mode. Figure 5A shows the sample area where the Raman mapping was performed to get the signal from ZOT and PEG with 10 mW power and 532 nm excitation wavelength. Corresponding mapping images of the same location show the distribution of ZnO in the composite sample in blue and PEG in red colour respectively. As can be observed, ZOT has been encapsulated with PEG as shown in the combined mapping image of the two components performed using confocal Raman provided with the instrument. Figure 5B shows the corresponding Raman spectra of the composite samples from 200 to 1350 cm^−1^. Using the instrument’s software, the spectra were corrected for the cosmic ray and background.

The signals from the two components (ZnO and PEG) have been deconvoluted using the software provided with the Instrument and the peaks have been identified using the database ‘ST Japan 5.2’. The peaks were found to match with the reports given in^32–36^. The inset of the Raman spectra shows the independent signals received for ZnO shown in blue, and PEG shown in light red. The main peaks identified for ZnO were present at 277, 331 and 437 cm^-1^ representing the first-order peaks for ZnO^22,26^. Another peak with a weak signal at ~ 1150 cm^-1^ corresponds to ZnO. The grating used for measuring the Raman spectra was 1800 gr/ mm, suitable for the ZnO Raman shifts. However, the spectra were taken for a shorter range of wavenumbers and then stitched together to get the complete spectra including the range for PEG. The Raman shifts for PEG were present at 362, 533, 580, 842, 858, 1061,1124, 1140, 1230 and 1279 cm^−1^ ^33–35,37^. Raman confirms the synthesis of composite samples. It is clearly shown, that a porous 3D ZOT + PEG hybrid network has been formed by the applied process. In the following section, the photocatalytic responses of fabricated 3D ZOT-PEG composites are reported in brief to verify the excellent catalytic functionality.

### Photocatalytic response of 3D ZOT-PEG composites

**Figure 6:**
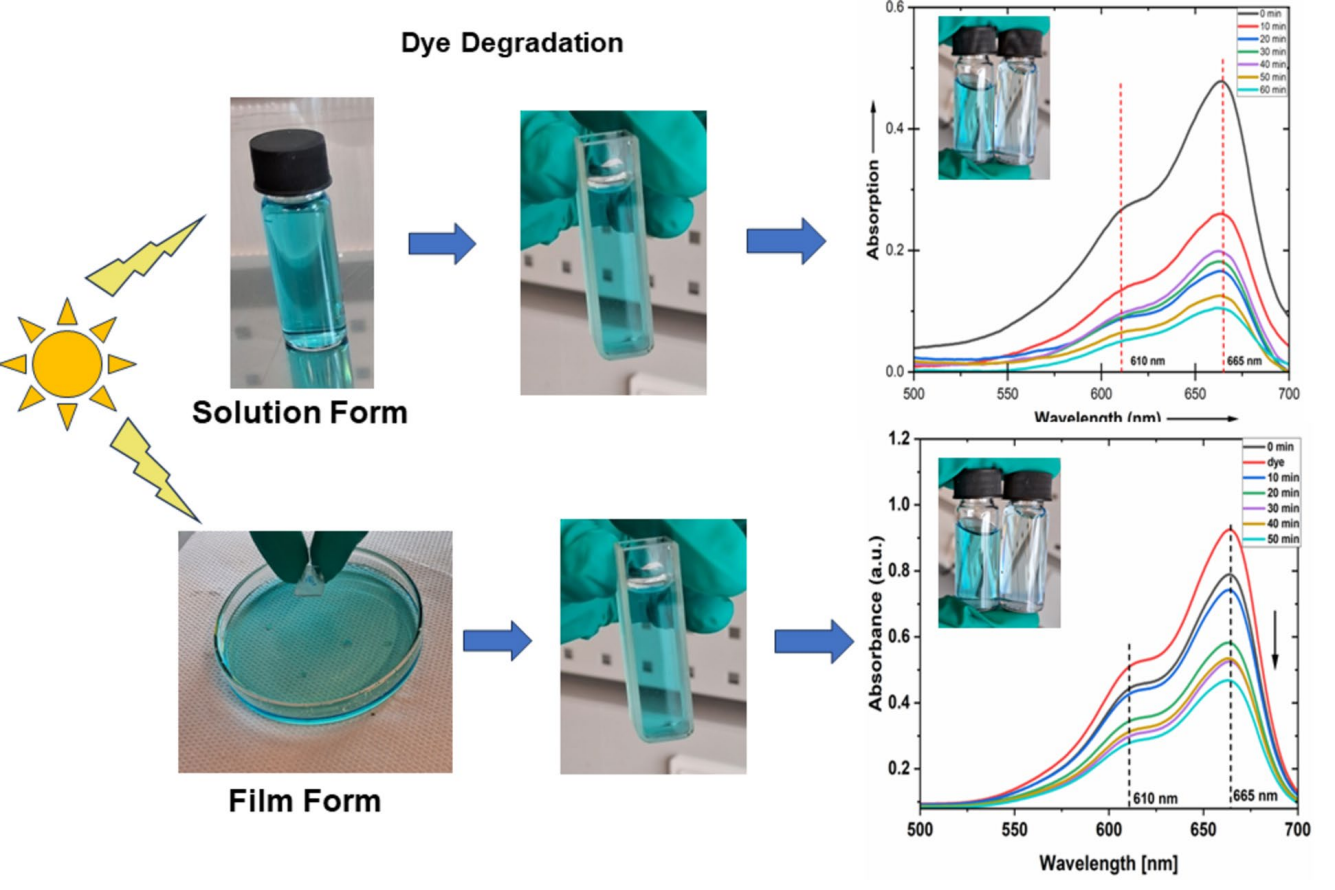
Dye degradation performance for the ZOT-PEG composite solution and ZOT-PEG composite film samples as catalyst.

The synthesized composite samples (solution and films) were studied for the photocatalysis activities for the degradation of MB dye, as represented in the schematic in Fig. 6.

The MB dye poses harmful effects on the aquatic life as they reduce the transparency of water and hinders the penetration of sunlight essential for their survival due to their complex aromatic compounds^2^. Thus, it is essential to find solutions towards breaking down of this harmful dye. The photocatalytic activity of (ZOT-PEG) specimen prepared in deionized water was investigated for the degradation of MB dye added as a contaminant in deionized water using optical absorption spectroscopy for different UV exposure time (up to 1 h with 10 min exposure time) to monitor the degradation rate for 10 µL solution of dye (0.1 mM) mixed with 5 mL of catalyst solution (2.5%) diluted in deionized water. The identical solution sample was exposed to UV light in steps of 10 min to keep the studies uniform, and absorption spectra were recorded in the quartz cuvette after each exposure cycle.

**Figure 7:**
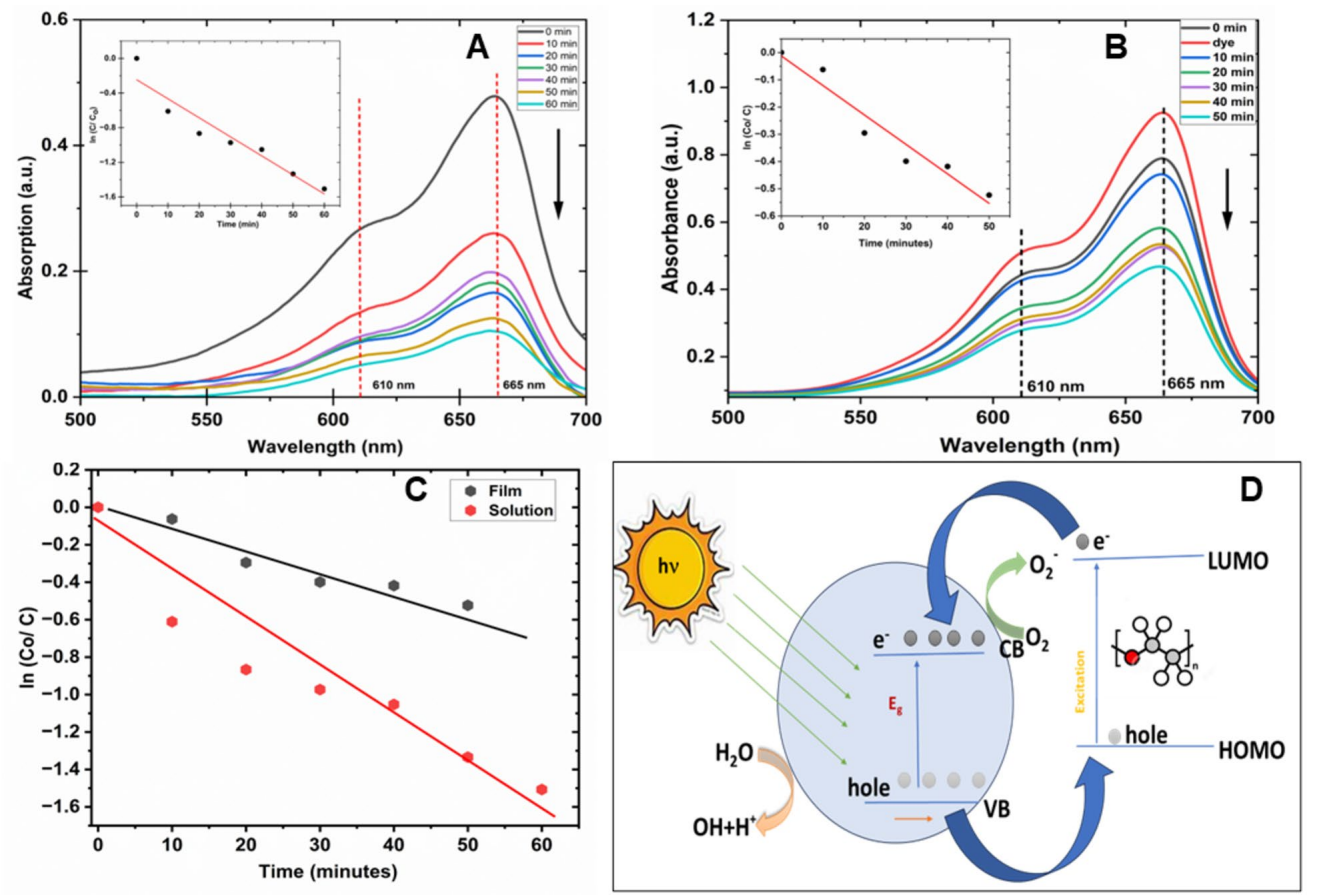
Absorption spectra of 0.1 mM methylene blue dye showing a photocatalytic reduction in the presence of composite material: (**A**) solution mixed in deionized water; (**B**) with thin film sample; with corresponding degradation rate shown in the insets; (**C**) Dye degradation rates in the presence of catalyst in the form of solution and film and (**D**) Schematic representation of photocatalytic mechanism with composite material.

Figure 7 shows the absorption spectra of MB dye exhibiting photocatalytic degradation in the presence of the catalyst for different UV light exposure times. The absorption peak of MB blue is exhibited at ~ 665 nm with a shoulder at ~ 610 nm. The inset confirms the rate of degradation estimated from the absorption spectra (shown in Fig. 7A). It can be seen that absorption intensity decays with exposure time indicating the degradation of the dye molecules^38^. The black curve corresponds the reference curve without any exposure to UV light. The intensity of the curves drops down with exposure time with respect to UV light. The kinetics of the dye degradation is described by Eq. (2):

2 $$\ln \left( {\frac{{{\varvec{Co}}}}{{\varvec{C}}}} \right) = {\varvec{kt}}$$

where, C_0_ is the initial concentration and C is the concentration at time t of the dye, and k is the rate constant.

The concentration of the undegraded dye was estimated from the absorption intensities and the rate constant was determined by the slope of the plot of ln(C_0_/C) versus time shown in the inset of Fig. 7A. The slope was found to be 0.022 ± 0.003, indicating the rate of dye degradation ~ 0.022 per minute. The dye degradation investigations were performed for the film samples: 5 mL of the same dye solution (0.1 mM) was taken in 5 Petri dishes with the composite films dipped into the solution and exposed to UV light at different times. The absorption spectra were consequently measured for each of the dye solution transferred into the quartz cuvette. The spectra are plotted for their degradation behavior as shown in Fig. 7B. The absorption intensity decreases with exposure time, indicating degradation of the dye molecules. The slope of the plot of log (C_0_/ C) versus time was found to be 0.011 ± 0.001 thus, the degradation rate was found to be ~ 0.011 per minute. The comparison of the two results (solution and film) is shown in the plot in Fig. 7C. As expected, the films show a slower rate of degradation which is expected since the contact area of the sample with the dye is small. However, the encouraging degradation is evident from the study as it doesn’t leave any harmful traces in the water and thus, makes it efficient for environmental remediation. A comparative table is provided below for the degradation of other harmful dyes by other groups in Table 2. It has been observed that the ZnO composites are capable of removing the harmful effects of synthetic dyes from the water and the efficiency for the photocatalytic actions may be improved by adding materials such as metals, polymers, plants extract etc.^3^ to answer the problem of fresh water supply thus, plays major role for the environmental remediation.

**Table 2:** Comparison table for the values of ZnO nanostructures based composites used for dye degradation.

S.no	Catalytic materials	Materials/dye	Photocatalytic degradation rate	Refs.
1	PEG functionalized ZnO nanoparticles	MB	0.0103 per minute (zero order)	^31^
2	N-doped ZnO nanocomposites	MB and RhB	98.11% and 86.21% respectively, after 3 h of sunlight irradiation	^39^
3	ZnO microtubes	MB	0.126 per min	^40^
4	Chitosan/TiO_2_/Glycerol Films in 1.4 cm of Water	*Near Wash Yellow Dye*	0.0256/ min	^41^
5	Zn@ZnO/PEG	Rhodamine B	0.003/ min	^42^
6	ZnO NPs with roselle flower and oil palm leaf extract	Methyl Orange & MB	0.8675/ h	^38^
			1.0784/ h	
			1.9213/ h	

## Discussion

With the lack of fresh water supplies available for the sustainability of humans due to the contaminants being thrown into the natural water resources, a lot of pressure is put on the global water supply and thus, threatens the biodiversity and the supply of fresh water for food production and other human needs^43^. Fresh drinking water is scarcity already in few of the countries with over one billion people not provided with adequate drinking water^44^ and thus, it has become one of the sustainable goals for better and green future^45^. Although, water is considered a renewable resource due to the natural water cycle in which the evaporated water is again come back to earth in the form of rain and refilling the natural water resources, but due to the unfriendly behavior of human being, this fresh water is being contaminated very fast^46^. Consequently, there is a need to find solution which will not only make the contaminated water fresh by removal of heavy metal contaminants or organic water pollutants, but also do not leave traces within the cleanup processes^47,48^. Catalysis are getting a lot of attention in this direction and provide an environmental friendly solution to pollutants abatement in water purification^49^ through removal of harmful organic dyes^50^. Various nanoparticles have been used as catalysts in waste water treatment; however, they result in problem of leaching from their surfaces which may result in bigger problem in near future^51^. Zinc oxide is one of the most studied material for environmental remediation and shows good photocatalytic degradation of organic compounds with high selectivity^52^ through an indirect pathway involving hydroxyl radicals as the oxidizing agents^38^.The PEG is a conducting polymer and corresponds to the same energy levels as ZnO. Thus, on exposure to UV or visible light, photogenerated charge carriers will be formed and transferred efficiently^31,53^.

The as-synthesized ZnO tetrapods capped with PEG reported here should show a higher photocatalytic activity because of their unique structure, which has a more active surface area for the binding of organic (dye) molecules. Above all, the tetrapodal 3D geometry makes it more porous and efficient in the dye degradation. When exposed to UV light, the conduction band electrons (*e*_*CB*_^−^) and valence-band holes (*h*_*VB*_^+^) are generated on the surface of ZnO tetrapods capped with PEG. The generated holes, reacted with PEG and water adhering to surfaces of ZnO to form highly reactive hydroxyl radicals (OH) which are responsible for the degradation of organic dye^10,54^, as shown in the schematic view in Fig. 7D. Molecules of MB were activated by light to an excited state which activates oxygen to yield oxidizing radicals (OH^*^)^39^. The photocatalytic superiority of ZOT could be attributed to the presence of a large specific area leading to the enhancement in dye adsorption, transportation, and light harvesting. The PEG is able to promote the charge-transfer in the polymer composite material by promoting the electron donor and hole acceptor mechanisms due to the presence of C and O atoms to facilitate the photocatalytic reaction^53^. Both the materials are UV sensitive and thus, the e-hole recombination was reduced. The mechanism of dye degradation in the presence of composite (ZOT-PEG) is given below:

3 $$ZnO \, + \, h\nu \to e^{ - } + \, h^{ + }$$

4 $$PEG \, + \, h\nu \to e \, + \, h^{ + }$$

5 $$e^{ - } + \, O_{2 } \to O_{2}^{* - }$$

Oxidation reaction:

6 $$h^{ + } + methylene \, blue \, dye \to CO_{2}$$

7 $$h^{ + } + \, H_{2} O \to {}^{*}OH \, + \, H^{ + }$$

Reduction reaction:

8 $$^{*} OH \, + \, methylene \, blue \, dye \to CO_{2}$$

The above mechanism shows the dye was degraded successfully, due to an increase in charge transfer rates, which resulted in a drastic reduction of the direct recombination of the photogenerated electron/ hole pairs^10,31,55–57^, essential to enhance the photocatalytic efficiency in the degradation of the dye molecule. Thus, the fabricated composite resulted in an excellent photocatalytic performance. Therefore, the dye molecule is broken into CO_2_ and H_2_O and thus, reducing water contamination, thus, does not result in formation of any secondary hazardous products^2^. The presented work demonstrated a significant method, with improved performance, for more efficient environmental remediation.

## Conclusion

This study synthesized a novel 3D polymer composite with zinc oxide tetrapods and polyethylene glycol for the photocatalytic degradation of harmful organic dyes. The FTIR and Raman spectra confirmed the formation of the ZOT-PEG composite with a sharp spectrum in Raman indicating the crystalline nature of the ZnO and PEG respectively. The overall length of ZOTs was found to be in between 10 to 50 µm. The EDX and Helium Orion microscopy confirmed the capping of PEG over ZOT structures. The fabricated 3D composites have been tested for degradation of organic dye, methylene blue, when mixed with deionized water (0.1 mM) as contaminant in the presence of composite material in the solution and film forms using absorption spectroscopy for different exposure times of UV light. The improved degradation was obtained for higher UV exposure time, demonstrating a good photocatalytic response with the degradation rate as ~ 0.022/ minute and 0.011/ minute, respectively for the solution and the film. With simple fabrication process and large-scale production possibility, these presented novel 3D ZOT-PEG composites with promising photocatalytic response could offer the potential platform alternative for wastewater treatment leading to significant implications in environment remediations for sustainable ecosystem.

## Data Availability

The datasets used and/or analysed during the current study available from the corresponding author on reasonable request.
